# Genetic and Hormonal Control of Bone Volume, Architecture, and Remodeling in XXY Mice

**DOI:** 10.1002/jbmr.104

**Published:** 2010-04-07

**Authors:** Peter Y Liu, Robert Kalak, YanHe Lue, Yue Jia, Krista Erkkila, Hong Zhou, Markus J Seibel, Christina Wang, Ronald S Swerdloff, Colin R Dunstan

**Affiliations:** 1 Division of Endocrinology, Department of Medicine, Harbor-UCLA Medical Center and Los Angeles Biomedical Research Institute (LA BioMed) at Harbor-UCLA Medical Center Torrance, CA, USA; 2 Endocrine and Metabolic Research Program, Woolcock Institute of Medical Research, University of Sydney Sydney, Australia; 3 Bone Biology Research Program, ANZAC Institute of Medical Research, University of Sydney Sydney, Australia; 4 Hospital for Children and Adolescents, University of Helsinki Helsinki, Finland; 5 Department of Biomedical Engineering, University of Sydney Sydney, Australia

**Keywords:** aneuploidy, klinefelter syndrome, XXY, testosterone, bone, x-inactivation, osteoporosis

## Abstract

Klinefelter syndrome is the most common chromosomal aneuploidy in men (XXY karyotype, 1 in 600 live births) and results in testicular (infertility and androgen deficiency) and nontesticular (cognitive impairment and osteoporosis) deficits. The extent to which skeletal changes are due to testosterone deficiency or arise directly from gene overdosage cannot be determined easily in humans. To answer this, we generated XXY mice through a four-generation breeding scheme. Eight intact XXY and 9 XY littermate controls and 8 castrated XXY mice and 8 castrated XY littermate controls were euthanized at 1 year of age. Castration occurred 6 months prior to killing. A third group of 9 XXY and 11 XY littermates were castrated and simultaneously implanted with a 1-cm Silastic testosterone capsule 8 weeks prior to sacrifice. Tibias were harvested from all three groups and examined by micro–computed tomography and histomorphometry. Blood testosterone concentration was assayed by radioimmunoassay. Compared with intact XY controls, intact androgen-deficient XXY mice had lower bone volume (6.8% ± 1.2% versus8.8% ± 1.7%, mean ± SD, *p* = .01) and thinner trabeculae (50 ± 4 µm versus 57 ± 5 µm, *p* = .007). Trabecular separation (270 ± 20 µm versus 270 ± 20 µm) or osteoclast number relative to bone surface (2.4 ± 1.0/mm^2^ versus 2.7 ± 1.5/mm^2^) did not differ significantly. Testosterone-replaced XXY mice continued to show lower bone volume (5.5% ± 2.4% versus 8.1% ± 3.5%, *p* = .026). They also exhibited greater trabecular separation (380 ± 69 µm versus 324 ± 62 µm, *p* = .040) but equivalent blood testosterone concentrations (6.3 ± 1.8 ng/mL versus 8.2 ± 4.2 ng/mL, *p* = .28) compared with testosterone-replaced XY littermates. In contrast, castration alone drastically decreased bone volume (*p* < .001), trabecular thickness (*p* = .05), and trabecular separation (*p* < .01) to such a great extent that differences between XXY and XY mice were undetectable. In conclusion, XXY mice replicate many features of human Klinefelter syndrome and therefore are a useful model for studying bone. Testosterone deficiency does not explain the bone phenotype because testosterone-replaced XXY mice show reduced bone volume despite similar blood testosterone levels. © 2010 American Society for Bone and Mineral Research.

## Introduction

Chromosomal dysjunction occurs during meiosis in all mammalian species and sometimes can result in aneuploidal live-birth offspring. Klinefelter syndrome (KS) is the most frequent sex chromosome aneuploidy in human males, occurring in 1 in 500 male conceptions, of which only five-eights to one-half progress to full term because the corresponding incidence in live male births is 1 in 800 to 1000.(1–3) The karyotype responsible is usually XXY,(4,5) although higher-order aneuploidies (XXXY and higher) can occur and are associated with more extreme phenotypes.(6)

KS is typified in all species examined to date (including rodents, Siberian tigers, and Australian kangaroos) by reproductive dysfunction expressed as androgen deficiency, small testicular size, and infertility. Additional nonreproductive features such as tall stature and osteoporosis are also characteristic in humans but occur with early onset of or untreated hypogonadism from any cause.(6–8) On the other hand, certain behavioral, neurologic, psychiatric, and cognitive deficits are specific to KS(9–13) and provide proof of principle that some features of KS cannot be mediated solely by androgen deficiency.

The features and consequences of human KS now have been identified without ascertainment bias through national population-based studies that link cytogenetic diagnoses with mortality registries or hospital admissions.(10–13) These studies confirm an increased risk of osteoporosis(13) and death from femoral fracture,(11) particularly in later life.(14) Furthermore, it has long been assumed that osteoporosis and increased fracture risk occur owing to coexisting androgen deficiency rather than KS per se, and many studies have not separated KS from other causes of hypogonadism analytically. Consistent with this hypothesis, both higher concurrent systemic testosterone concentrations(15) and shorter androgen receptor polyalanine length (which results in greater androgen receptor transactivation)(16) are associated with higher bone mineral density (BMD) in men. Testosterone therapy administered to men with primary hypogonadism (including those with KS) also generally improves BMD,(17) but normalization of bone may be incomplete in some men with KS,(18,19) especially if therapy is suboptimal.(20) Furthermore, men with KS exhibit other bone and joint abnormalities (ie, premature fusion and excessive calcification of coronal sutures,(21) abnormal joint development,(22) and osteoarthritis(13)) that are not typically observed in other hypogonadal men.

For these reasons, we hypothesized that the osteoporosis exhibited by KS men may not arise exclusively from androgen deficiency and that genes, including those located on the X chromosome that escape X inactivation, could be partly responsible. Establishing the existence of such genes eventually could unveil novel pathways important for bone architecture, volume, or turnover. However, separating hormonal from genetic causes requires interventional studies that cannot be undertaken systematically in humans. We therefore developed an XXY mouse model that exhibits androgen deficiency and impaired learning, analogous to the hormonal, testicular, and cognitive phenotype of human KS.(23) The purpose of this study was to characterize bone architecture, volume, and turnover in intact, castrated, and simultaneously castrated and testosterone-replaced XXY mice by static and dynamic histomorphometry, micro–computed tomography (µCT), and dual-energy X-ray absorptiometry (DXA).

## Materials and Methods

### Animals

Breeding pairs of C57BL/6J XY* male and XX female mice were initially purchased from the Jackson Laboratory (Bar Harbor, ME, USA). XXY mice (41,XXY) and their littermate XY mice (40,XY) were produced in the fourth generation from our breeding colony, as described previously.(23) Two separate groups of intact (XXY *n* = 8, XY littermate controls *n* = 9) and castrated (XXY *n* = 8, XY littermate controls *n* = 8) mice were generated and euthanized at age 12 to 15 months. The second group was castrated 6 months prior to killing. A third group of littermates (XXY *n* = 9, XY *n* = 11) was simultaneously castrated and implanted with a 1-cm Silastic capsule of testosterone 8 weeks prior to killing at age 14 to 34 months under standard isofluorane anesthesia, as described previously by us.(24) A subgroup (XXY *n* = 6, XY *n* = 7) of this third group of mice was injected with calcein (Sigma, Castle Hill, Australia) solution (30 µg/kg of body weight) intraperitoneally 13 and 3 days prior to killing to allow dynamic histomorphometry and also was scanned by DXA (Hologic 4500A using the small-animal software module, Bedford, MA, USA) at the time of killing to examine whole-body composition and bone density.

The animal breeding colony was established and housed in a standard animal facility, 3 to 4 animals per cage, under controlled temperature (22°C) and photoperiod (12 hours light/dark) with free access to water and mouse chow. Animal breeding, handling, and experimentation were in accordance with the recommendations of the American Veterinary Medical Association and were approved by the Harbor-UCLA Biomedical Research Institute Animal Care and Use Review Committee.

### Tissue collection and specimen preparation

At euthanization, tibias were harvested for histologic and µCT evaluation, and plasma was collected by cardiac puncture for later testosterone measurement. Tibias were dissected and fixed in 4% formalin, buffered with 0.1 mol/L of phosphate buffer (pH 7.4) for 24 hours at 4°C, and then stored in 70% ethanol for µCT evaluation. After µCT analysis, tibias were decalcified with 10% EDTA and embedded in paraffin. Serial 5-µm sections were stained with hematoxylin and eosin (H&E) or fast green and safranin-O for general histologic evaluation. To identify osteoclasts, sections were stained for tartrate resistant acid phosphatase (TRACP) using naphthol AS–BI phosphate (Sigma) as a substrate and fast red violet Luria-Bertani salt (Sigma) as the detection agent.

### µCT anaylsis

µCT of tibias was performed using a Skyscan 1172 scanner (SkyScan, Kontich, Belgium). Scanning occurred at 100 kV and 100 µA with a 1-mm aluminium filter, and the exposure time was set to 590 ms. In total, 1800 projections were collected for each tibia at a resolution of 6.93 µm/pixel. Sections were reconstructed using a modified Feldkamp cone-beam algorithm with beam-hardening correction set to 50. VGStudio MAX 1.2 software (Volume Graphics GmbH, Heidelberg, Germany) produced 3D visualizations from reconstructed sections. Trabecular and cortical morphometry of proximal tibias were quantified with CTAnalyser software (Version 1.02, SkyScan). The volume of interest for trabecular bone was selected within the endosteal borders from 0.1 to 1.1 mm below the distal surface of the proximal growth plate. Trabecular morphology was described by bone volume fraction [BV/tissue volume (TV)], trabecular number (Tb.N), trabecular separation (Tb.Sp), trabecular thickness (Tb.Th), bone surface to bone volume ratio (BS/BV), and trabecular pattern factor (Tb.Pf), which is an index of trabecular connectivity (higher values of Tb.Pf correspond with lower connectivity).(25) Transverse sections of tibial cortical bone were analyzed at a level 20% by length below the proximal end of the tibia corresponding to approximately 3 mm from the growth plate.

### Histomorphometry

Histomorphometric analysis of the proximal tibial metaphysis was conducted in all mice. Measurements were performed on 5-µm sections stained with TRACP, H&E, or fast green and safranin-O using the Bioquant Osteo II System (Bioquant, Nashville, TN, USA). The region of interest was a 1 × 1 mm area located 0.1 mm below the growth plate of the tibia. Osteoclast number and osteoclast surface relative to bone surface were measured with osteoclasts identified as TRACP^+^ (red-stained) multinucleated cells in direct apposition to bone surfaces. Osteoblasts were identified by their cuboidal morphology, and osteoblast surface was quantified.

### Fibroblast culture and karyotype analysis

Standard karyotyping was performed on cultured fibroblasts obtained from ear clips in adult mice as described previously.(23) Briefly, a 1- to 2-mm^2^ section of tissue was dissected from an ear clipping. The sample was minced and digested with 1.25% trypsin (Gibco, Invitrogen Co., New York, NY, USA) for 30 minutes, followed by collagenase (Gibco Invitrogen) for an hour and half at 37°C. The dispersed cells were suspended in Amino-Max-II medium (Gibco, Invitrogen), which supports the growth of anchored fibroblast cells. The cells were placed in flasks and cultured for 5 to 7 days at 37°C in a CO_2_ incubator. Once appropriate colony formation was observed, KaryoMAX Colcemid solution (Gibco, Invitrogen) was added to stop mitotic division. The cultured fibroblasts were harvested after a minor digestion with trypsin-EDTA solution (Gibco, Invitrogen). The harvested cells were suspended in 0.075 M potassium chloride solution (Gibco, Invitrogen) and incubated in a water bath at 37°C for 20 minutes, fixed in a mixture of methanol and acetic acid (3:1 methanol–acetic acid), spread on clean glass slides, and air dried for fluorescence in situ hybridization (FISH) analysis. Images were examined with a Zeiss fluorescence microscope (Göttingen, Lower Saxony, Germany) using Image-Pro Plus software (Silver Spring, MD, USA).

### Testosterone assays

Plasma was stored at −20°C for later measurement of testosterone concentrations by RIA using a kit from DPC (Coat-a-Tube, Torrance, CA, USA), as reported previously.(23) The minimal detection limit of the assay was 0.25 ng/mL, and the intra- and interassay coefficients of variations (CV) were approximately 10%.

### Statistical analysis

Five comparisons were planned: (1) intact XY versus intact XXY, (2) castrated XY versus castrated XXY, (3) castrated and testosterone-implanted XY versus castrated and testosterone-implanted XXY, (4) intact XY versus castrated XY, and (5) intact XXY versus castrated XXY. Student's *t* tests evaluated all comparisons, except comparison 3 because age is known to alter bone biology. For this analysis, two-way ANOVA models with age (≤15 months and >15 months) and karyotype (XY versus XXY), and the interactions were constructed. Where appropriate, models without the interaction term were constructed to confirm full factorial findings. Means and standard errors of the means (SEM) are shown unless otherwise stated. For comparisons 1, 2, and 3, *p* < .05 was considered statistically significant because all comparisons were independent and performed in separate sets of mice. For comparisons 4 and 5, a more stringent *p* < .025 was considered statistically significant using Holm's method to sequentially correct for multiple comparisons.(26) All statistical analyses were performed using SAS Proc Ttest and Mixed (SAS Version 9.1, SAS Institute, Cary, NC, USA).

## Results

### Plasma testosterone concentrations

Plasma hormone concentrations directly confirmed equivalent systemic testosterone exposure in XY and XY mice after castration and then after simultaneous castration and testosterone replacement. We have shown previously that plasma testosterone concentration in intact XXY mice is less than that in XY mice (*p* < .05): 2.6 ± 0.8.1 ng/dL (*n* = 7) and 5.7 ± 0.8.1 ng/dL (*n* = 7).(23) Plasma testosterone concentrations after castration were undetectable. Plasma testosterone concentrations after simultaneous castration and testosterone implantation were increased to intact adult male mice levels and equivalently: 6.3 ± 0.7 ng/dL and 8.2 ± 1.6 ng/dL in XXY (*n* = 6) and XY (*n* = 7) mice, respectively (*p* = .28).

### Bone architecture

**Fig. 1:**
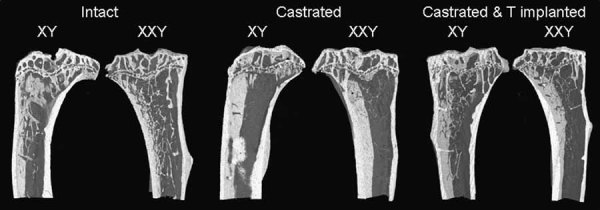
Illustrative longitudinally orientated proximal tibial sections obtained from 3D reconstructed serial µCT sections. Micrographs from intact (*left*), castrated (*center*), and simultaneously castrated and testosterone-treated (*right*) XY and XXY mice are shown. Castration results in profound loss of bone after castration in both XY and XXY mice (*center*).

**Fig. 2:**
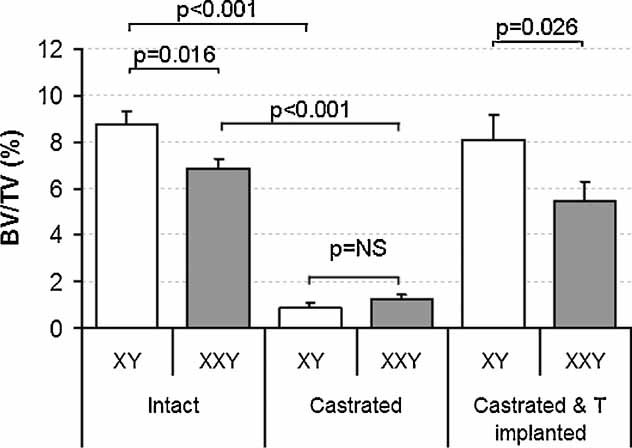
Tibial bone volume fraction in intact (*left*), castrated (*center*), and simultaneously castrated and testosterone-treated (*right*) XY and XXY mice. XXY mice are osteopenic compared with XY mice in the intact state (*p* = .016) as well as when simultaneously castrated and testosterone-replaced (*p* = .026). Castrated XXY and XY mice are equally (*p* = .30) and profoundly (*p* < .001 for both) osteopenic. Data are mean ± SEM.

Longitudinally orientated micrographs of 3D reconstructed proximal tibial images in intact, castrated, and testosterone-replaced XXY and XY mice are shown in Fig. 1. Profound loss of bone occurs after castration in both XXY and XY mice. Quantitative µCT findings (Table 1 and Fig. 2) confirm qualitative observations (Fig. 1). Intact XXY mice are osteopenic (show reduced bone volume, BV/TV) compared with XY mice, and they remain so even after 2 months of equivalent testosterone therapy (Table 1 and Fig. 2). This osteopenia is associated with reduced trabecular thickness in intact XXY mice and reduced trabecular number in testosterone-replaced XXY mice compared with littermate controls (Table 1). However, trabecular connectivity (Tb.Pf) did not differ according to karyotype in intact, castrated, or testosterone-replaced mice (Table 1). Six months of castration results in profound loss of bone so that architectural differences between XXY and XY mice are no long discernible. Castration markedly disrupts all aspects of trabecular architecture in XY and XXY mice, except trabecular thickness, which was relatively preserved in XXY (*p* = 0.91) and XY (*p* = .03; considered not significant after multiple comparison testing) mice.

**Table 1:** Tibial µCT Architecture

		Tibial trabecular bone												Tibial cortical bone			
			
		BV/TV (%)		Tb.N (1/mm)		Tb.Sp (µm)		Tb.Th (µm)		BS/BV (1/mm)		Tb.Pf (1/mm)		Area (mm^2^)		Thickness (µm)	
Intact	XY	8.8	(0.6)	1.56	(0.08)	272	(7)	57.0	(1.7)	67	(2.9)	22.8	(2.0)	0.86	(0.03)	127	(4.2)
	XXY	6.8	(0.4)	1.36	(0.05)	270	(8)	50.1	(1.3)	76	(2.6)	27.2	(1.4)	0.81	(0.03)	128	(4.9)
	Testosterone test XY versus XXY	0.016		0.062		0.876		0.007		0.025		0.095		0.207		0.814	
Castrated	XY	0.9	(0.2)	0.17	(0.03)	413	(28)	46.9	(4.5)	108	(13)	59.7	(7.1)	0.69	(0.02)	121	(7.1)
	XXY	1.2	(0.2)	0.25	(0.04)	399	(13)	49.7	(2.7)	103	(9.1)	53.7	(6.1)	0.74	(0.02)	129	(3.3)
	Testosterone test XY cast versus XXY cast	0.304		0.169		0.626		0.572		0.750		0.532		0.066		0.273	
Castrated and testosterone implanted	XY	8.1	(1.1)	1.30	(0.12)	324	(19)	60.4	(3.0)	66	(3.4)	23.4	(1.6)	0.72	(0.03)	96	(5.8)
	XXY	5.5	(0.8)	0.95	(0.13)	380	(23)	57.8	(2.8)	70	(3.1)	26.5	(1.7)	0.71	(0.04)	91	(7.5)
	ANOVA XY cast testosterone versus XXY cast testosterone	0.026		0.019		0.040		0.342		0.301		0.224		0.992		0.667	
	Testosterone test XY versus XYcast	0.000		0.000		0.000		0.030		0.003		0.000		0.000		0.473	
	Testosterone test XXY versus XXYcast	0.000		0.000		0.000		0.913		0.015		0.001		0.093		0.874	

*Note:* Data are mean (SEM). BV/TV is bone volume/tissue volume; Tb.N, Tb.Sp, Tb.Th, and TB.Pf are trabecular number, separation, thickness, and pattern factor, respectively; and BS/BV is bone surface/bone volume.

Cortical bone area and thickness in the tibias were similar between XXY and XY mice under all three experimental conditions (Table 1).

### Bone histomorphometry

No significant differences in osteoblast or osteoclast surface in relation to bone surface were detected between XXY and XY mice under any of the three experimental conditions (intact, castrated, and simultaneously castrated and testosterone-replaced; Table 2). No differences in bone apposition rates were observed (*p* = .35).

**Table 2:** Tibial Histomorphometry

		Ob.S/BS (%)		Oc.S/BS (%)		N.Oc/BS (1/mm)	
Intact	XY	8.6	(1.6)	9.7	(1.8)	2.67	(0.61)
	XXY	12.2	(3.1)	10.5	(2.0)	2.43	(0.39)
	Testosterone test XY versus XXY	0.301		0.776		0.743	
Castrated	XY	9.5	(2.0)	16.3	(3.0)	4.79	(0.8)
	XXY	14.9	(3.5)	16.5	(3.3)	3.71	(0.75)
	Testosterone test XY cast versus XXYcast	0.251		0.962		0.352	
Castrated and testosterone implanted	XY	4.2	(0.7)	2.8	(0.8)	1.39	(0.34)
	XXY	1.7	(1.1)	1.9	(0.4)	1.58	(0.39)
	Testosterone test XY cast versus XXY cast	0.083		0.44		0.352	
	Testosterone test XY versus XY cast	0.713		0.087		0.061	
	Testosterone test XXY versus XXYcast	0.570		0.155		0.173	

Note: Data are mean (SEM). Ob.S/BS and Oc.S/BS are obsteoblast and osteoclast surface relative to bone surface, respectively; N.Oc/BS is osteoclast number relative to bone surface.

#### DXA scans

**Fig. 3:**
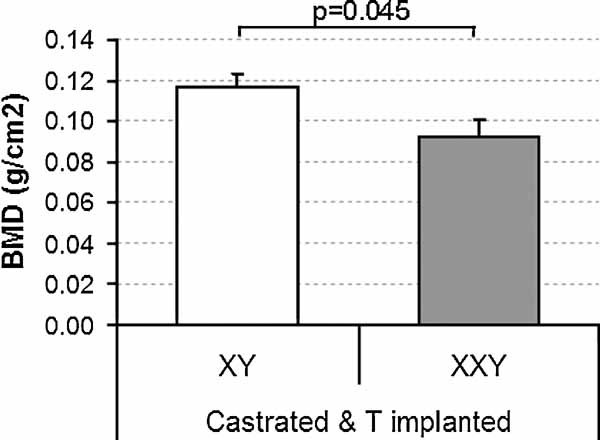
BMD measured by DXA in castrated and simultaneously castrated and testosterone-treated XY and XXY mice. XXY mice are significantly osteopenic (*p* = .045). Data are mean ± SEM.

DXA scans (Fig. 3) show a significant (*p* = .045), persistent reduction in total bone in the entire bodies of XXY mice after 2 months of matched testosterone therapy and confirm the findings shown by µCT of the tibia (Figs. 1 and 2). Total, lean, and fat mass did not differ significantly between XXY and XY mice (all *p* > .2) under testosterone-replaced conditions.

## Discussion

In this study we demonstrate that mice with an XXY genotype present with an osteopenic phenotype, a similar finding to that observed in men with KS. The reduction in bone mass in these XXY mice may be due in part to impaired gonadal function. Indeed, orchidectomy of normal and XXY mice produce profound reductions in bone mass in both genotypes, reflecting the potency of androgens/estrogens in maintaining bone mass. However, XXY mice orchidectomized and concurrently testosterone replaced with implanted pellets for 8 weeks had persistent reduced bone mass relative to that in similarly treated XY littermates. Although we do not know whether the duration of the testosterone treatment was optimal in rodents, these latter results suggest a role for genotype independent of gonadal function in determining bone mass in XXY mice.

Here we provide preliminary evidence that X-linked genes that escape inactivation may be responsible for reductions in bone mass. Further work is needed, but a systematic approach to identify these genes is possible because the DNA sequence of the X chromosome is known.(27) Genome-wide linkage studies also have associated BMD and area bone size with Xq27,(28,29) and DNA expression sequence tags, including a number with hypothetical protein products, that escape X-inactivation have been identified.(30) Altogether, we provide proof of principle that studying chromosomal aneuploidies is a useful strategy to identify novel genes, as has been postulated for understanding the genetic basis of psychosis.(31)

A systematic and exhaustive examination of bone architecture in any XXY mammal has not been performed previously. Here we show that (intact) adult XXY mice have osteopenia and that this is due to trabecular, not cortical bone changes. The predominate change is reduced trabecular thickness rather than reduced trabecular separation or number, and this is important because reduced trabecular number appears to reduce bone strength more than reduced trabecular thickness.(32) Furthermore, the thinning rather than loss of trabeculae is suggestive but not conclusive of decreased bone formation.(33) Nevertheless, bone histomorphometry did not unveil the cellular basis for the reduction in trabecular thickness because osteoblast and osteoclast surface areas were each comparable with those of XY littermate controls. The lack of difference in static cell measurements between XXY and XY mice may reflect altered cell function (rather than cell appearance or number). Alternatively, the cellular changes leading to bone loss may have occurred earlier during skeletal development and may no longer be apparent in mature mice.(34)

Orchidectomy in XXY and XY mice resulted in marked loss of bone and profoundly disrupted trabecular architecture to a similar extent in both genotypes. Trabecular separation was increased, whereas trabecular number and connectivity were decreased (each *p* ≤ .001). However, trabecular thickness was not changed (*p* = .91). These results highlight the importance of androgens and estrogens in maintaining bone mass because bone loss was so profound with steroidal withdrawal that additional genotypic effects on bone mass may have been overwhelmed.

In a strategy to remove the effect of differences in gonadal function on bone changes in XY and XXY mice, we conducted in a study in which XXY and XY mice were orchidectomized but immediately received a testosterone implant. Simultaneous orchidectomy and testosterone replacement in XXY mice unveiled persistent osteopenia associated with reduced trabecular number and increased trabecular separation but preserved trabecular thickness compared with identically treated XY littermate mice. This testosterone-replacement strategy resulted in matched testosterone levels comparable with adult XY mice and was applied for 2 months. The changes in trabecular architecture differ from those seen in intact XXY mice, and this is consistent with our contention that the bone phenotype of XXY mice is partly but not completely due to androgen deficiency. Histomorphometric analysis failed to identify differences in bone formation (double tetracycline labeling) or in osteoblast or osteoclast surfaces between XXY and XY mice in the intact, orchidectomized, or orchidectomized and testosterone-replacement studies. The reason for this is unclear but could be due to establishment of a new steady state in bone turnover at the time of euthanasia in these mice. Nevertheless, osteopenia per se in the testosterone-treated whole animal was confirmed independently by DXA. Importantly, our DXA findings show equivalent total, lean, and fat mass between hormonally matched XXY and XY mice. These data provide some reassurance that the bone findings do not relate to changes in body composition, physical activity and bone loading, net aromatase action, growth hormone, or adipokines,(35) although we did not measure the latter directly.

We did not show any difference in cortical bone in intact, castrated, or simultaneously castrated and testosterone-replaced mice. Trabecular bone mass is typically a more sensitive indicator of developing osteoporosis (which is a cause of osteopenia), but the reason for the maintenance of cortical bone mass despite loss of trabecular bone is unclear, although cortical and trabecular bone loss may be subject to different genetic control in mice.(36) Such an explanation would be consistent with a genetic component to bone loss in XXY mice. Androgen effects are exerted mostly on trabecular bone, whereas aromatization to estrogens is also important for cortical bone,(37) although not exclusively so.(34,37) This effect on trabecular bone is mediated by androgen receptors located in mature osteoblasts that are responsible for bone mineralization.(34) However, not all studies agree, and overexpression of androgen receptor actually may decrease endocortical bone formation.(38) The reasons for these discrepancies may relate to ontogeny.

In conclusion, we provide evidence, using a mouse model, that the osteopenia of KS is not due to just androgen deficiency and that there is an additional effect of genotype. If this evidence can be translated to human KS, it would provide a rationale for the difficulty in normalizing bone mass through androgen therapies in these patients. Perhaps of greater importance, these finding encourage further studies that seek to identify X-linked genes that could contribute to bone loss in KS patients and that would have broader relevance to osteoporotic patients in general.
